# Social language in autism spectrum disorder: A computational analysis of sentiment and linguistic abstraction

**DOI:** 10.1371/journal.pone.0229985

**Published:** 2020-03-06

**Authors:** Izabela Chojnicka, Aleksander Wawer

**Affiliations:** 1 Department of Health and Rehabilitation Psychology, Faculty of Psychology, University of Warsaw, Warsaw, Poland; 2 The Linguistic Engineering Group, Institute of Computer Science Polish Academy of Sciences, Warsaw, Poland; Istituto di Fisiologia Clinica Consiglio Nazionale delle Ricerche, ITALY

## Abstract

Individuals with autism spectrum disorder (ASD) demonstrate impairments with pragmatic (social) language, including narrative skills and conversational abilities. We aimed to quantitatively characterize narrative performance in ASD using natural language processing techniques: sentiment and language abstraction analyses based on the Linguistic Category Model. Individuals with ASD and with typical development matched for age, gender, ethnicity, and verbal and nonverbal intelligence quotients produced language samples during two standardized tasks from the Autism Diagnostic Observation Schedule, Second Edition assessment: *Telling a Story from a Book* and *Description of a Picture*. Only the narratives produced during the Book Task differed between ASD and control groups in terms of emotional polarity and language abstraction. Participants with typical development used words with positive sentiment more often in comparison to individuals with ASD. In the case of words with negative sentiment, the differences were marginally significant (participants with typical development used words with negative sentiment more often). The Book Task narratives of individuals with ASD were also characterized by a lower level of language abstraction than narratives of peers with typical development. Linguistic abstraction was strongly positively correlated with a higher number of words with emotional polarity. Neither linguistic abstraction nor emotional polarity correlated with participants’ age or verbal and nonverbal IQ. The results support the promise of sentiment and language abstraction analyses as a useful tool for the quantitative, fully automated assessment of narrative abilities among individuals with ASD.

## Introduction

Social and communication deficits are a core clinical feature of autism spectrum disorder (ASD; [1, 2]). Language delay and child’s speech and communicative development are among the most frequent initial parental concerns [3]. Regardless of heterogeneity in the language capacities among individuals with ASD, impairments with pragmatic (social) language, including discourse skills and conversational abilities are observed across the autism spectrum [4]. Individuals with ASD have difficulties with to-and-fro conversation, including problems with initiating and sustaining conversational interactions. People with ASD rarely offer personal information spontaneously and express less interest in others’ ideas, experiences and emotions [5].

Pragmatic language impairments among individuals on the autism spectrum include narrative (i.e., storytelling) difficulties [6]. Bruner describes narration as an ability of thinking, communicating and sharing experiences as a way to adjust and reconstruct them in order to better understand them [7]. Narrative discourse is a complex cognitive task involving the integration of information, remembering certain details of the story and the use of gathered knowledge about the world to produce a coherent narrative describing a series of actions and events that unfold over time.

Previous research on storytelling and ASD examined differences between narratives produced by people on the autism spectrum and people with typical development (TD). Storytelling as a vulnerable domain in autism spectrum disorder seems to be universal across languages with different typologies (e.g., [8–11]). Individuals with ASD produce narratives with fewer number of words and utterances as well as reduced lexical diversity (number of different words) than children and adolescents with TD [12, 13]. Narratofives produced by individuals with ASD are less causally connected and less coherent than those produced by controls [14]. With regard to the syntactic complexity of the narration in ASD, the studies yielded contradictory results. Some results indicated differences in morphosyntactic trajectory between individuals with ASD and peers with TD [13, 15]; others did not reveal such differences [14]. Another characteristic thoroughly studied in narratives of individuals with ASD is the internal state language (ISL), i.e., the vocabulary used to describe character perceptions, emotions and thoughts. In most studies, they are verbs and adjectives referring to the characters’ emotional (e.g., “to laugh”, “happy”) and cognitive (e.g., “to know”, “confused”) states [6]. Previous studies have shown relation between mental language in narratives and Theory of Mind abilities [13]. The results of meta-analysis by Baixauli et al. [6] indicate that participants with ASD use fewer ISL terms than controls, with IQ acting as a moderator variable: individuals with high IQ levels present more significant difficulties in comparison to their peers with TD with the verbal description of internal states.

To date, the vast majority of research on narrative discourse in ASD has applied hand-coding methods. With advances in natural language processing (NLP) techniques, computational approaches have become available for studying narrative competence in ASD. The use of natural language processing and other computational techniques to capture language features in a quantitative way might not apply to everyone on the autism spectrum, e.g., children at early stages of language development, or non-verbal people with ASD. However, it might help in the future to identify subtle characteristics of the disorder difficult to capture for a parent or a clinician during a short interaction that would support screening and the diagnostic process of children with high-functioning ASD.

Previous studies that have used computational linguistic models to characterize narrative performance in ASD applied semantic word models (e.g., [16, 17]). Lee et al. [16] and Losh and Gordon [17] used Latent Semantic Analysis (LSA) and showed that narratives produced by individuals with ASD were significantly lower in semantic similarity to the “gold standard text” [16] or default semantic space [17] than controls. LSA is a quantitative tool which identifies the semantic similarities of words through patterns of their statistical co-occurrence within a “semantic space” (the meaning of a word is a point in a vector space). LSA can also be used to measure the shared semantic meaning between bodies of text. In the study of semi-structured conversations, produced by individuals with ASD during Autism Diagnostic Observation Schedule (ADOS) assessment, Goodkind et al. [18] applied vector semantic models (meaning of each word is represented by its vector, such that semantically similar words are mapped to proximate points in a multidimensional space; representation of each document is a sum of vectors for each of its words) and demonstrated that semi-structured conversations produced by individuals with ASD were semantically further from a conversational sample by two children with TD collected during the ADOS and designated as a “gold standard”, and there was more variability within the ASD group than within the TD group.

Both semantic word models and LSA are based on low-rank vector approximations of text utterances. In these methods one can not interpret the vector elements that are associated with autism. Our motivation to apply dictionary methods was exploratory: contrary to semantic word models and LSA, dictionary approaches are based on nameable and psychologically informative dimensions of language. One can hypothesize that similar properties of language were used by vector models (e.g., LSA), but it can not be easily explained.

### Sentiment analysis and linguistic abstraction

Here we applied another approach to analyze narratives produced by individuals with autism spectrum disorder during two standardized tasks from the Autism Diagnostic Observation Schedule, Second Edition (ADOS-2) diagnostic tool: *Telling a Story from a Book* and *Description of a Picture*. We focused on the sentiment and language abstraction analyses based on the Linguistic Category Model (LCM), both applicable to quantify psycholinguistic properties of texts.

#### Linguistic category model

LCM, referred to as the metasemantic model of interpersonal language, makes it possible to identify the nuances of how people use interpersonal terms when they are describing social events in communication [19]. LCM typology is a well-established tool to measure language abstraction. Differences in the level of language abstraction can be observed even when people describe the same events. A person can describe an event in a concrete way, presenting visible behaviors and details. Another person may provide interpretative narration, presenting subjective descriptions of people, behaviors and elements that are not directly visible such as feelings and traits [20].

LCM is based on categorizing verbs reflecting their level of abstraction. The principal distinction is made between three verb categories: Descriptive Action Verbs (DAVs), Interpretative Action Verbs (IAVs), and State Verbs (SVs; [21]). While Action Verbs (DAVs and IAVs) have a clearly defined beginning and end, SVs do not. DAVs are the most concrete verbs used to describe a single, observable event (e.g., ‘A kicks B’). IAVs describe specific observable events (e.g., ‘A hurts B’) and are considered to be more abstract than DAVs as they do not preserve the perceptual features of an action. The most abstract category is State Verbs that refer to mental (e.g., to think, to understand) and emotional (e.g., to admire) states or changes therein. LCM principles [21] describe two criteria for differentiating DAVs and IAVs. Firstly, DAVs, unlike IAVs, have at least one physically invariant feature (e.g., to kick–leg, to kiss–mouth). Secondly, IAVs, in contrast to DAVs, have an evaluative component (e.g., positive–to help, to encourage; negative–to cheat, to bully). Although DAVs are inherently neutral (e.g., to push), they can gain an emotional aspect depending on the context (e.g., push someone in front of a bus vs. push someone away from an approaching bus).

The LCM was developed to apply to the terms used in describing individuals and their behavior according to the level of verb abstraction. All the verb categories form an ordering from DAV to IAV to SV, according to their increasing abstraction. As in [22]: “as one moves from DAVs […], subject informativeness increases, situative informativeness decreases, and the sentence appears more endurable, less verifiable, and more likely to be the object of disagreement or dispute”.

The first LCM-based dictionary was developed for English, but recently a Polish language version of LCM was published [23]. Characterizing language variation using LCM was successfully applied to several psychological phenomena, including linguistic intergroup bias, perpetuation of stereotypes [24] and personality differences [25]. The application of the LCM to atypically developing populations as pursued in our paper is a novel usage.

#### Sentiment analysis

Sentiment analysis is a natural language processing technique that uses computational algorithms to extract subjective information from text in terms of the positive or negative emotional tones of the statement. Sentiment analysis is a widely researched subject; some good overviews of its sub-fields and currently applied techniques, along with many further references, can be found in [26, 27] and for the Polish language specifically in [28]. However, it has yet to be explored in the field of autism spectrum disorder or other neurodevelopmental disorders.

Sentiment analysis is based on algorithms that use natural language processing to categorize text pieces as positive, neutral, or negative in order to determine the emotional tone the text carries. One approach to this is by counting occurrences of words with positive or negative meaning, such as ‘enthusiastic’, ‘beautiful’, ‘violence’, ‘terrible’, but also words with weaker emotional polarization, such as ‘home’. Most of the current work in sentiment analysis is carried out in domain-dependent fashion (restricted to specific types of language or text) using machine learning or deep learning [27]. In this approach, algorithms learn from data sets restricted to selected topic and text type (e.g., movie reviews or opinions about particular products) and reach high accuracy when applied to texts of the same topic and type. However, this method fails to be universal, as the accuracy of other topics and types of texts is usually low. Moreover, it is focused on categorizing whole texts and does not enable the amount of positive and negative words to be quantified. For these reasons, we used a more widely applicable method based on an open-domain (universal) dictionary of sentiment.

Research to date that relied mostly on hand-coding methods has provided valuable, broad characterizations of narrative difficulties across different chronological and mental ages in ASD [6]. However, manual coding of results has its drawbacks: it is labor-intensive and time-consuming, subjective and requires extensive training for coders to achieve adequate reliability [16, 17]. Computational linguistic measures may have the ability to overcome these constraints. The approach we used includes measuring the vocabulary associated with internal state language. In addition to detecting verbs and adjectives as in traditional ISL measurement methods, our approach embodies nouns with emotional polarization (sentiment analysis) and the level of utterance abstraction (LCM). Moreover, as opposed to the subjective, manual counting of ISL terms, our approach is fully automated.

In the present study, we compared the narratives of participants with TD and participants with ASD during two standardized tasks from the ADOS-2 Modules 3 and 4 assessments. We applied sentiment and language abstraction analyses which have not yet been applied in ASD. We hope that the results obtained add to the knowledge related to narration discourse in ASD, and ISL in particular, which until now has only been analyzed using hand-coding methods. For our assessment, we focused on the three main research questions:

Q1: Are individuals with ASD less likely to use words with emotional polarity than peers with TD? We predicted, that participants with ASD use both fewer words with negative and positive sentiment.

Q2: Are the narratives produced by individuals with ASD characterized by a lower level of abstraction compared to narratives of peers with TD? We hypothesized that the level of utterance abstraction and number of state verbs would be lower for narratives produced by individuals with ASD.

Q3: Does the speech of individuals with ASD differ, in terms of inflectional characteristics and grammatical categories, from the speech of participants with TD? The last question is exploratory in nature.

## Materials and methods

### Participants

Participants included 25 individuals with idiopathic ASD (ASD Group) and 25 controls with typical development (TD Group) matched for age, gender, ethnicity, verbal and nonverbal intelligence quotients (Table 1). Inclusion criteria for participants were (1) age ≥ 7 years, (2) non-verbal IQ ≥ 80, (3) Polish as a first and primary language, (4) no hearing, sight, and mobility impairments, (5) for ASD Group–clinical diagnosis of an autism spectrum disorder determined by a psychiatrist based on ICD-10 diagnostic criteria and meeting criteria for autism spectrum on the ADOS, ADI-R and SCQ. The exclusion criteria for controls included a personal or family history of ASD, history of developmental disorders, neurological, or psychiatric conditions or suspected developmental problems.

**Table 1:** Demographics of the sample.

	ASD Group (*n* = 25)	TD Group (*n* = 25)	p-value
Males *n*	22	22	
Chronological age in years *M (SD)*	14.55 (5.46)	14.38 (5.83)	.68
Chronological age range	7.1–24.6	7.3–25.3	
Nonverbal IQ *M (SD)*	109.08 (13.04)	114.64 (12.50)	.98
Nonverbal IQ range	80–140	80–134	
Verbal IQ *M (SD)*	108.28 (18.30)	113.12 (14.33)	.24
Verbal IQ range	72–131	85–141	

ASD–autism spectrum disorder; TD–typical development; *M*–Mean; *SD*–standard deviation; IQ–Intelligence Quotient.

### Procedure

The project was approved by the Faculty of Psychology, University of Warsaw Research Ethics Committee. Informed consent was signed prior to participation in the study by: (a) the parents of participating children under 16 years of age, (b) the parents of participating children aged 16 and older and the participants themselves.

Diagnosis of each participant in the ASD Group was confirmed using gold standard diagnostic instruments: (a) the Autism Diagnostic Interview-Revised [29, 30], (b) Autism Diagnostic Observation Schedule, Second Edition–Modules 3 and 4 designed for verbally fluent individuals [5, 31], as well as (c) Social Communication Questionnaire [32]; Polish adaptation by Pisula et al., 2019, unpublished manuscript, and excluded, using the above-mentioned tools, for participants from the TD Group. The intellectual functioning of all participants was tested using the Wechsler Intelligence Scale for Children-Revised for verbal children and adolescents aged 6.0–16.11 [33] and the Wechsler Adult Intelligence Scale for verbal participants older than 16.11 [34].

#### Tasks

Language samples were derived from the ADOS-2, a standardized, semi-structured observation schedule for diagnosing individuals on the autism spectrum [5]. The participants’ narratives were recorded and then transcribed by two experienced, trained transcribers, following the Codes for Human Analysis of Transcripts (CHAT; [35]). Transcribers were blind to group status and were trained to greater than 80% reliability. One of the trained transcribers revised prepared transcripts and resolved any discrepancies based on the audio-recordings.

*ADOS-2 Telling a Story from a Book task*. The picture book ‘Tuesday’ by David Wiesner during the *Telling a Story from a Book* ADOS-2 task (the Book Task) was used in order to elicit narratives. The book depicts the adventures of frogs which one Tuesday evening begin to float on the leaves of water lilies and fly to the town. Pictures include unreal and humorous elements, and the characters experience different mental and emotional states.

Following ADOS-2 instructions, the examiner introduced the story at pages 1 and 2: “Let’s look at this book. It tells a story in pictures. See, it starts on Tuesday evening, around eight o’clock in the evening. A turtle is sitting on a log. He sees something. Can you tell me the story as we go along?” Then the participant continued the story. During this task an examiner is allowed to interact with the participant to maintain rapport and elicit narratives as naturally as possible. If a participant does not respond, an examiner is allowed to say “I wonder what happens next” (no more than two prompts). An examiner should not model labeling emotions. In our experiment, the examiners did not take turns in telling a story.

*ADOS-2 Description of a Picture task*. A picture of a tropical holiday resort was used during the *Description of a Picture* ADOS-2 task (the Picture Task) in order to elicit spontaneous language samples. Participants were asked to describe what was happening in the picture. The picture portrays a number of people who spend their time in various ways (sunbathing, sailing, swimming, etc.) during their holidays at a seaside resort. While describing the picture, participant may tell what is happening on it using short sentences or produce longer descriptions and short stories of what is happening in a given situation or to a given character.

Following ADOS-2 instructions, the examiner introduced the task by saying “Let’s look at this picture now. Can you tell me about it? What is happening here?” During this task an examiner is allowed to interact with the participant, but should not provide substantive information about the picture and should refrain from modeling or asking specific questions about particular parts of the picture.

#### Analysis techniques

In order to automatically analyse the language samples, we employed a fully automated NLP pipeline. For each input text, it produces a vector containing variables that describe the frequencies of inflectional (morphosyntactic) classes of words contained in the text, as well as sentiment and abstraction. The vector contains nearly 80 variables with over 70 resulting from morphosyntactic description.

The architecture of the pipeline is a standard one for morphologically rich languages such as Polish. It consists of two connected modules:

1. Morphosyntactic analyser Morfeusz. Its main task is to assign possible lemmas and morphosyntactic features to each word or token in a text. Technically, it is performed by a dictionary compiled into the form of finite state automaton [36]. Morfeusz also performs tokenization, which is the process of splitting input strings into tokens and punctuation marks. For each input word, Morfeusz outputs a number of interpretations along with their lemmas and morphological tags that describe their possible morphosyntactic roles in a sentence.

2. Morphosyntactic disambiguation module Concraft-pl. It aims to resolve morphosyntactic ambiguities that arise in Morfeusz by using contextual, sentence-level information. In the Polish language, multiple word forms may have different part-of-speech and other morphosyntactic tags depending on their context (including lemmas). This is somewhat analogous to the English language in which the word book may be either a verb (”to book a room”) or a noun (”to read a book”) and it is necessary to analyze the sentence to determine which interpretation is correct. The goal of this module is to use morphological analysis results as input and select appropriate interpretations according to the context. Concraft-pl uses a conditional random fields (CRFs) algorithm, adapted to tagging highly-inflectional languages such as Polish [37]. Our research uses this module to find word lemmas and frequency counts of morphological tags and grammatical classes, and to find sentiment-carrying vocabulary and LCM labels for verbs.

The pipeline we used contains modules that communicate via a Thrift framework, which is an environment for cross-language services that enables services implemented in varying programming environments to efficiently and seamlessly inter-operate. The implementation we used has been put together as a part of the Multiservice NLP toolbox [38].

To compute word sentiment, we used a dictionary of 3276 lemmas, of which 1494 words are positive and 1774 negative. It has been manually compiled as a part of the Sentipejd tool [39]. Lemma-level sentiments avoid the issues raised by word sense disambiguation errors and are more universal than sense-level sentiment dictionaries. The lemmas in this dictionary were compared to morphologically disambiguated lemmas provided for input texts by the Concraft-pl module.

To compute LCM tags, we used the dictionary described in [23]. It consists of three parts: (1) the most frequent 4000 Polish verb senses with sense-level LCM labels, (2) another 1200 verbs with lemma-level annotation, and finally (3) all other remaining Polish verbs with automatically inferred LCM labels. To disambiguate verb senses, we used the Wosedon word sense disambiguation tool provided by CLARIN-PL web services (https://ws.clarin-pl.eu/wsd.shtml).

**Fig 1:**
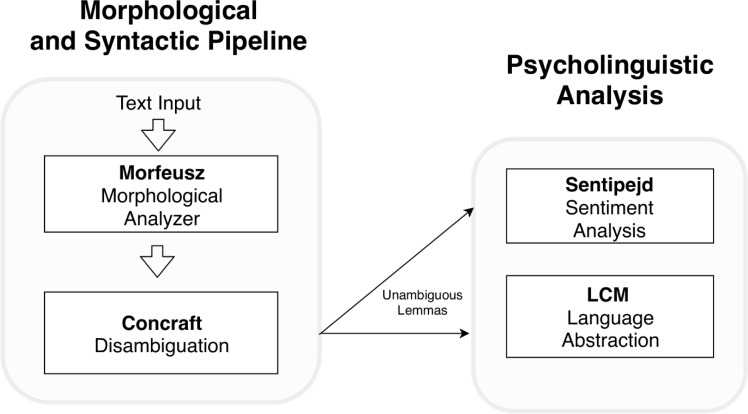
Diagram illustrating the pipeline used for natural language processing.

Elements of the NLP pipeline are illustrated in the diagram presented in Fig 1.

Table 2 illustrates outputs provided by each NLP module for the sample Polish sentence “Ala ma radosny nastrój” (which translates to “Ala is in a good mood”). For each input token, Morfeusz produces several possible interpretations, each with a different set of morphosyntactic tags and different lemmas. The choice of the proper interpretation (lemma and tags) is performed by Concraft, which produces probability scores from 0 to 1. Finally, LCM verb types are assigned.

**Table 2:** Illustration of the outputs of each NLP module.

	Morfeusz		Con-craft	Sentipejd	LCM
Input tokens PL| EN	Lemmas (PL | EN)	Morphosyntactic Tags	Probability	Sentiment	Verb Type
Ala | Ala	Ala	subst:sg:nom:f	0.89	-	-
	Al	subst:sg:gen:acc:m1	0	-	-
	Alo	subst:sg:gen:acc:m1	0	-	-
ma | has	mój | mine	adj:sg:nom.voc:f:pos	0	-	-
	mieć | have	fin:sg:ter:imperf	1.0	-	SV
radosny | joyful	radosny	adj:sg:acc:m3:pos	1.0	positive	-
		adj:sg:nom.voc:m1.m2.m3:pos	0	-	-
nastrój | mood	nastrój	subst:sg:nom.acc:m3	1.0	-	-
	nastroić	impt:sg:sec:perf	0	-	-

acc—accusative case; f–feminine gender; fin–verb in the non-past form; gen—genitive case; impt–verb in the imperative mood; imperf–imperfective aspect; nom—nominative case; m1—masculine personal gender; m2—masculine animal gender, m3—inanimate masculine gender; perf—perfective aspect

pos—positive degree; sec—second person; sg—singular number; subst—substantive (noun); ter–third person; voc—vocative case; LCM—Linguistic Category Model; SV—State verb; EN–English, PL—Polish

Polish is a highly inflected language with several lexemic and flexemic classes. Lexemes are minimal meaningful units of language (e.g., verbs, nouns, pronouns, etc.) and considered as sets of flexemes. Flexeme is a set of wordforms which inflect in a uniform way (e.g., infinitives, adjectival participles, etc.). We analyzed Polish lexemes and flexemes according to a morphosyntactic tagset in a large-scale corpus of Polish being developed at the Institute of Computer Science, Polish Academy of Sciences [40, 41].

For each participant, the number of tokens (an individual occurrence of a linguistic unit) of a given word form (i.e., words with positive sentiment, negative sentiment, DAVs, IAVs, SVs, grammatical classes and categories) in the whole task was added up. The level of utterance abstraction was calculated according to the weighted summation formula: DAV+IAV*2+SV*3 [21]. Authors of the LCM recommend it to calculate the level of abstraction of any given utterance.

#### Statistical approach

In order to answer the first two research questions (whether individuals with ASD are less likely to use words with emotional polarity and whether the narratives produced by individuals with ASD are characterized by a lower level of abstraction compared to narratives of peers with TD) we conducted independent samples t-tests and non-parametric Mann-Whitney U tests, when the assumptions for the t-test regarding normality of data distribution and homogeneity of variance were not met. We compared the duration of the tasks, mean utterance length, the number of words with positive sentiment, with negative sentiment, the number of DAVs, IAVs and SVs, as well as the level of utterance abstraction. We calculated Cohen’s d and r statistics to measure effect sizes. Pearson correlations tested relationships between sentiment words and the level of language abstraction, and age, verbal and nonverbal IQ.

A two-way mixed ANOVA with repeated measures was performed to compare differences between groups on levels of language abstraction and words with emotional polarity in both ADOS-2 tasks.

Subsequently, we were looking for an answer to the third question: whether the speech of individuals with ASD differs, in terms of inflectional characteristics and grammatical categories, from the speech of participants with TD. For each textual utterance, we obtained from the automated NLP pipeline a frequency of nearly 70 possible types of words and word forms, grouped into grammatical classes and categories, described by a system of morphosyntactic tags. This typology includes part-of-speech, but is more fine-grained as the grammatical classes are induced from inflection patterns. Morphosyntactic tags contain information related to verb conjugation (changing of the form of a word according to tense, person, number and other verb categories specific to Polish), noun and adjective declension (changing according to grammatical case, gender, number) and so on. For more information about the morphosyntactic tagset for Polish and its principles see [40]. Again, we conducted independent samples t-tests and non-parametric Mann-Whitney U tests. As we did not have a pre-established hypothesis for these comparisons, we applied Bonferroni adjustments [42].

## Results

### Book Task

#### Duration of the task and number of tokens

There were no statistically significant differences between TD and ASD Groups in relation to the duration of the task (*p* = .49) and the total number of tokens (*p* = .107). However, we received statistically significant results for the comparison of the number of tokens per one single statement (*p* < .001, Cohen’s *d* = .46). Participants with ASD produced shorter utterances than controls (*M* = 8.37, *SD* = 6.94 for ASD Group and *M* = 9.78, *SD* = 7.50 for TD Group, Cohen’s *d* = .46).

#### Sentiment analysis

Participants from the TD Group statistically more often used in their statements words with a positive evaluative meaning in comparison to individuals with ASD (for the whole task *M* = .84, *SD* = .75 and *M* = 2.28, *SD* = 2.41 for ASD and TD Groups respectively, *t(48)* = 2.86, *p* = .008, Cohen’s *d* = .83). In the case of words with negative sentiment, the differences were marginally significant(*M* = 1.08, *SD* = 1.38 and *M* = 2.32, *SD* = 2.78 for ASD and TD Groups respectively, *t(48)* = 2.00, *p* = .054, Cohen’s *d* = .58).

#### Language abstraction analysis

**Fig 2:**
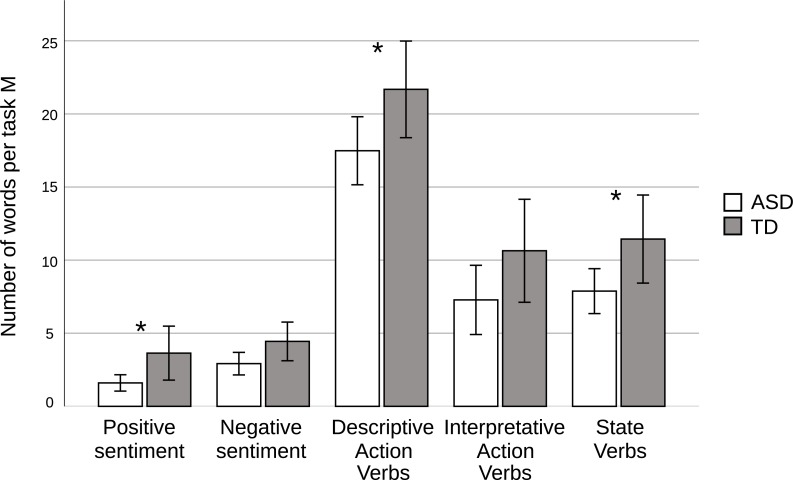
Group differences in the number of words used in sentiment and linguistic abstraction analyses for participants with autism spectrum disorder (ASD) and controls with typical development (TD); * *p* < .05.

Statistically significant differences were obtained for the State Verbs (*t(48)* = 2.15, *p* = .037, Cohen’s *d* = .62) and Descriptive Action Verbs (*t(48)* = 2.32, *p* = .025, Cohen’s *d* = .67). Individuals with ASD were less likely to use both DAVs (*M* = 13.24, *SD* = 7.48 for the ASD Group, *M* = 18.48, *SD* = 8.48 for the TD Group), and SVs (*M* = 13.00, *SD* = 7.35 for the ASD Group, *M* = 18.36, *SD* = 10.04 for the TD Group) in comparison to typically developing controls. There were no statistically significant differences for Interpretative Action Verbs (*p* = .144). We also obtained statistically significant differences between the two groups when analyzing the level of utterance abstraction in accordance with the DAV + IAV * 2 + SV * 3 formula (*t(48)* = 2.42, *p* = .020, Cohen’s *d* = .70). The level of utterance abstraction was higher among participants with TD (*M* = 94.12, *SD* = 42.48) than participants with ASD (*M* = 68.24, *SD* = 32.64). Linguistic abstraction was positively associated with higher number of words with positive sentiment (*r* = .657, *r*^*2*^ = .43, *p* < .001) in the whole sample and by group (*r* = .493, *r*^*2*^ = .24, *p* = .012 and *r* = .751, *r*^*2*^ = .56, *p* = .003 for participants with ASD and TD, respectively). Linguistic abstraction was also positively correlated with higher number of words with negative sentiment in the whole sample (*r* = .621, *r*^*2*^ = .39, *p* < .001) and by group (*r* = .577, *r*^*2*^ = .33, p < .001 and *r* = .623, *r*^*2*^ = .39, *p* < .001 for participants with ASD and TD, respectively). Neither linguistic abstraction nor emotional polarity correlated with participants’ age or verbal and nonverbal IQ. The number of words with sentiment orientation, DAVs, IAVs and SVs among ASD and TD groups is shown in Fig 2.

#### Analysis of morphosyntax

We performed analyses for nearly 70 possible types of words and word forms, grouped into grammatical classes and categories, described by a system of morphosyntactic tags that contain information related to verb conjugation (changing of the form of a word according to tense, person, number and other verb categories specific to Polish), noun and adjective declension (changing according to grammatical case, gender, number) and so on.

Significant statistical differences were obtained for several types of morphosyntactic tags. However, after Bonferroni correction the differences were no longer statistically significant.

### Picture task

There were no statistically significant differences between TD and ASD Groups in relation to the total number of tokens (*p* = .68) and the mean utterance length (*p* = .69). However, participants with ASD needed more time to complete the task (*M* = 160.71 seconds, *SD* = 89.96) than controls (*M* = 99.11, *SD* = 48.46, *U* = 115.00, *Z* = -2.085, *p* = .037, *r* = .29).

In contrast to the Book Task, in the case of the Picture Task, no statistically significant differences were found between individuals with ASD and peers with TD with reference to the language abstraction and words with emotional polarity. Linguistic abstraction was positively correlated with a higher number of words with positive sentiment (*r* = .522, *r*^*2*^ = .27, *p* < .01), but not with words with negative sentiment (*r* = .133, *r*^*2*^ = .02, *p* = .433). Neither linguistic abstraction nor emotional polarity correlated with participants’ age or verbal and nonverbal IQ.

A two-way mixed ANOVA with repeated measures was performed to compare differences between groups (ASD and TD) on levels of language abstraction and words with emotional polarity in the Picture and in the Book Tasks. Mauchly's test showed the data met the assumption of sphericity for all comparisons. For the linguistic abstraction analysis, there was a significant main effect of the task (*F(1*, *37)* = 73.231, *p* < .001, η2 = .677). There was neither a significant main effect of the group (*p* = .093) nor significant interaction between group and task (*p* = .149). Similarly, for the words with negative sentiment, there was a significant main effect of the task (*F(1*, *37)* = 53.147, *p* < .001, η2 = .617). There was not a significant main effect of the group (*p* = .238) nor significant interaction between group and task (*p* = .275). Bonferroni's post hoc tests showed a significantly lower level of language abstraction (*p* < .001) and fewer words with negative sentiment (*p* < .001) for the Picture Task than for the Book Task. An analysis of the number of words with positive sentiment revealed that there was not a significant main effect of the task (*p* = .222) nor group (*p* = .091) and there was no interaction between group and task (*p* = .222).

## Discussion

Previous studies that used detailed hand-coding schemes provided valuable insights into narrative discourse skills in autism spectrum disorder (e.g., [6, 13, 14]). In the current study we attempted to apply natural language processing techniques, i.e., sentiment analysis, language abstraction analysis and analysis of morphosyntax in order to characterize language samples and narratives among individuals with high-functioning ASD and controls with typical development. Until now, neither sentiment nor language abstraction has been used in studies of individuals with autism. Furthermore, we postulate that analyzing narratives in a morphologically rich language such as Polish is of value because of differences in morphosyntactic complexity, including many types of words and word forms related to verb conjugation as well as noun and adjective declension.

We analyzed language samples produced during two standardized tasks from the ADOS-2 assessment: *Telling a Story from a Book* and *Description of a Picture*. Interestingly, only the narratives produced during the Book Task differed between ASD and controls. We received no statistically significant results for the Picture Task. A two-way mixed ANOVA with repeated measures revealed significant differences between both tasks for the level of abstraction and number words with emotional polarity. During the Picture Task, participants used fewer words with negative sentiment and their utterances were characterized by a lower level of abstraction than during the Book Task. The Picture Task requires naming objects, interactions between people depicted in the picture, and the activities they perform, while the Book Task examines the ability to generate a narrative with the support of the pictures in the book about a sequential story. The Picture Task instruction includes the hierarchical sequence of social presses provided by the examiner that increase the degree of task structure if the general request to describe the picture causes difficulties for the participant. Our results indicate that not every pictorial stimulus may be equally good in order to differentiate the speech of people with ASD from the speech of peers with TD, particularly using natural language processing techniques. Prior evidence suggests that narrative context matters: participants with ASD exhibited more difficulties in less structured tasks, i.e., narratives of personal experiences or pictures from the Thematic Apperception Test [43, 44]. On the contrary, Baixauli et al. [6] reported no significant differences for the type of narrative used in several studies analyzed in their meta-analysis. However, they compared picture book/fictional storytelling vs. autobiographical/personal/everyday activities stories rather than a single picture description, as in the case of our study.

With regard to the narrative microstructure, utterances used by individuals with ASD were shorter than those produced by peers with TD in the Book Task, but not in the Picture Task. However, the summarized number of tokens used per task was comparable in both groups, both for the Book and the Picture Task. These results enable us to assume that we compared data sets of similar volumes.

According to Question 1, the Book Task narratives from ASD and TD Groups were quantitatively different in terms of the emotionally oriented words with large effect size for words with positive and medium effect size for words with negative polarity. Individuals with ASD used fewer words with positive (*p* = .008) and marginally fewer words with negative sentiment (*p* = .054). People with ASD have difficulty identifying, understanding, and describing emotions experienced by themselves and others [5]. The results we obtained show that narratives of individuals with autism spectrum disorder are less complex than of individuals with typical development not only in directly naming or describing emotions, but also in using other emotionally oriented words (e.g., trust, pain, wise, hilarious).

According to Question 2, the Book Task narratives of individuals with ASD were characterized by a lower level of language abstraction than narratives of participants from the TD Group (*p* = .020, medium effect size). The level of language abstraction was strongly, significantly, and positively correlated with words with emotional polarity. What is worth emphasizing, linguistic abstraction and sentiment were not associated with age, verbal, or nonverbal IQ. Participants with autism spectrum disorder used fewer State Verbs, that indicate mental or emotional states, than peers with TD. Moreover, individuals with autism spectrum disorder used fewer DAVs, the most concrete types of verbs in LCM typology, than individuals from the TD Group. This points to difficulties with the description of the story presented in the book not only at the interpretative (abstract) level, but also to convey specific, visible behaviors and details. This is consistent with the results of previous studies as narrative impairments are a central feature of autism spectrum disorder (e.g., [16]). People with ASD demonstrate considerable difficulties with narrative macrostructure, including a lower number of causal connections between events and deficits of narrative structure [6]. Individuals with ASD seem reluctant to narrate and tend to produce stories that lack coherence and the integration of protagonists’ thoughts and emotions [14, 44].

Finally, we tested whether the ASD Group’s Book Task narratives differ from the TD Group narratives in terms of inflectional characteristics and grammatical categories, including verbs, nouns, adjectives, adverbs, pronouns, numerals and others. We found no significant results indicating such differences. In prior work, the authors focused on difficulties with the understanding and use of grammar (grammatical errors) of some individuals with ASD and syntactic complexity (e.g., complex sentences, passive constructions, questions, clauses with non-canonical order) [6, 45] rather than morphological characteristics of language in ASD. Previous research yielded inconsistent results. Young et al. [46] reported no differences between participants with ASD and controls in terms of syntax and grammar. No differences in syntactic complexity were reported also by Diehl, Bennetto and Young [14] either. Baixauli et al. [6] concluded that results regarding indicators of grammar and syntactic complexity are relevant to a degree in that children are able to produce utterances that are long enough to present complex syntactic structures. Their results suggest delayed growth in some aspects of morphosyntactic acquisition among children on the autism spectrum. Regardless of the syntactic complexity, our results suggest no marked differences regarding types of words and word forms related to verb conjugation as well as noun and adjective declension in ASD.

Our study sample was gender biased (88% men) due to the gender disproportion in the population of people on the autism spectrum [47]. Most previous studies of narrative difficulties in ASD have been conducted in samples with a predominance of men. Therefore, prior results may not generalize to women on the autism spectrum. In one recent study, Boorse et al. [48] found that the previous observation that children with ASD use fewer cognitive process words is true for boys only, while language focused on objects is a sex-neutral linguistic marker of ASD.

Another limitation of our study is its wide age range of participants, given the long-time course of narrative development. However, meta-analysis by Baixauli et al. [6] revealed no differences in subgroups based on age. Given the relatively small sample size, we were not able to divide our sample into subgroups based on age or IQ level.

We analyzed narratives produced in Polish, which is a highly inflected language, with relatively free word order. Although there are many differences between Polish and English, they do not concern the aspects of communication analyzed in this work. There is a need for further research to replicate our findings with larger samples, considering age and intellectual abilities as potential moderators, other types of text samples and other languages, with particular attention to possible sex differences, as our study sample did not allow for gender comparisons.

Together, findings show that the sentiment and language abstraction analyses seem to be promising methods to characterize selected aspects of narratives in ASD. The observation that individuals with ASD use fewer words relating to mental and emotional states is not a novel finding [6]. However, the use of fully automated natural language processing techniques to capture this in a quantitative way is novel and in our opinion worth studying. A computational approach allows for large-scale empirical research and an in-depth characterization of large cohorts that could not be carried out using hand-coding methods, where the coding is made by a human expert. The results reported in this paper form the basis for future implementations of automated tools supporting clinicians and researchers working with ASD. Our study confirmed the usability of selected linguistic information (sentiment and language abstraction), but one may easily envision linking it with relevant non-linguistic variables to combine as an input for machine learning models related to ASD. These tools could be used to improve screening, diagnosis and intervention planning. Further studies are needed to investigate this, especially collecting multimodal and historical data.

This does not mean that models based solely on linguistic data are not worth investigating in the future. From a technical point of view, their applicability requires no more than a personal computer or smartphone. Data processing can be simplified even further by automatically transcribing the interviews using one of the existing speech-to-text (ASR) services. An overall solution like this, including an ASR, the NLP pipelines as described in our paper and machine learning models, could record the entire interview and display ASD-related measures to a researcher or clinician briefly after its completion.

In terms of clinical practice, as early as 2009, Demner-Fushman, Chapman, and McDonald [49] wrote about the possibilities of NLP applications in Computerized Clinical Decision Support that aims to help health care providers and the public make decisions by providing health-related information that is easily accessible at the time it is needed. We hope that our study takes us one small step further towards translating advances in the field of NLP into changes in ASD research and clinical practice in the future.

## Conclusions

The primary contribution of this study is to show that sentiment and language abstraction analyses distinguish individuals with ASD from peers with typical development: individuals with ASD used fewer words with emotional polarity, particularly with positive sentiment and their utterances were less abstract than the narratives of controls. This is a step toward developing a quantifiable and objective computational measure of language characteristics in ASD and other neurogenetic developmental disorders of language. Further research is needed to replicate these findings with other language samples and other types of narrative stimuli with attention to possible sex differences, age and IQ that can act as moderators and that we were not able to explore.

## Supporting information

S1 Data (XLSX)
